# Care of Babies

**Published:** 1870-09

**Authors:** 


### CARE OF BABIES.

ONE third of all the children born, die before they are two years old, and three fourths of these perish unnecessarily; perish as a consequence of the neglect or ignorance of mothers. Most infants are physicked and fed to death. No medicine whatever, not the modest "catnip tea," should be given to an infant without the direction of the family physician.

As to food, the practice is, the moment an infant is noticed to cry, it is fed; the result is, that in less than a week the little thing cries oftener from colic than from hunger, which may often be known by its vomiting soon after it is fed, or by its refusing to take food; the great, the essential point, is to feed all children at regular intervals; from neglect of this, infants are made dyspeptic before they are a month old, and between alternate feeding and physicking, they go off in convulsions, water on the brain, or diarrhœa. Notice at what intervals food is necessary, and feed only at such times, these being greater as the child gets older; next keep the child abundantly warm; keep it constantly clean; let it be in the open air every day, and never allow it to be showered or bathed in cold water.

